# Inhibition of Aspartate β-Hydroxylase Enhances Anti-Tumor Immunity

**DOI:** 10.2147/ITT.S530987

**Published:** 2025-07-07

**Authors:** Shweta Dilip Johari, Katerina Krausova, Barbora Zucha, Carlos Eduardo Madureira Trufen, Ingrid Polakova, Mark Olsen, Michal Smahel

**Affiliations:** 1 Department of Genetics and Microbiology, Faculty of Science, Charles University, BIOCEV, Vestec, Czech Republic; 2 Czech Centre for Phenogenomics & Laboratory of Transgenic Models of Diseases, Institute of Molecular Genetics of the Czech Academy of Sciences, Prague, Czech Republic; 3 Department of Pharmaceutical Sciences, College of Pharmacy – Glendale, Midwestern University, Glendale, AZ, USA

**Keywords:** cancer immunotherapy, ASPH, adaptive immunity, scRNA-seq, tumor microenvironment

## Abstract

**Purpose:**

Aspartate β-hydroxylase (ASPH) contributes to carcinogenesis by promoting tumor cell proliferation, migration, and invasion. The enzymatic activity of ASPH can be inhibited by small molecule inhibitors that have been shown to have anti-metastatic activity in rodent models. ASPH has also been shown to inhibit the activation of natural killer (NK) cells. Therefore, this study aimed to investigate the effect of ASPH inhibition on the induction of anti-tumor immunity and to analyze the immune cells involved.

**Methods:**

In the mouse TC-1/A9 model characterized by reversible downregulation of major histocompatibility class I (MHC-I) molecules, ASPH inhibition was combined with stimulation of innate and/or adaptive immunity, and the anti-tumor response was analyzed by evaluation of tumor growth, in vivo depletion of immune cell subpopulations, and ELISPOT assay. Characteristics of immune cells in the spleen and tumor were determined by flow cytometry and single-cell RNA sequencing (scRNA-seq).

**Results:**

ASPH inhibition did not reduce tumor growth or promote the anti-tumor effect of innate immunity stimulation with the synthetic oligonucleotide ODN1826, but it significantly enhanced tumor growth reduction induced by DNA vaccination. In vivo immune cell depletion suggested that CD8^+^ T cells played a critical role in this immunity stimulated by combined treatment with ASPH inhibition and DNA vaccination. ASPH inhibition also significantly enhanced the specific response of CD8^+^ T cells induced by DNA vaccination in splenocytes, as detected by ELISPOT assay, and reduced the number of regulatory T cells in tumors. scRNA-seq confirmed the improved activation of CD8^+^ T cells in tumor-infiltrating cells after combined therapy with DNA vaccination and ASPH inhibition. It also showed activation of NK cells, macrophages, and dendritic cells in tumors.

**Conclusion:**

ASPH inhibition stimulated T-cell-mediated adaptive immunity induced by DNA vaccination. Different types of lymphoid and myeloid cells were likely involved in the activated immune response that was efficient against tumors with MHC-I downregulation, which are often resistant to T-cell-based therapies. Due to different types of activated immune cells, ASPH inhibition could improve immunotherapy for tumors with various MHC-I expression levels.

## Graphical Abstract

**Figure:**
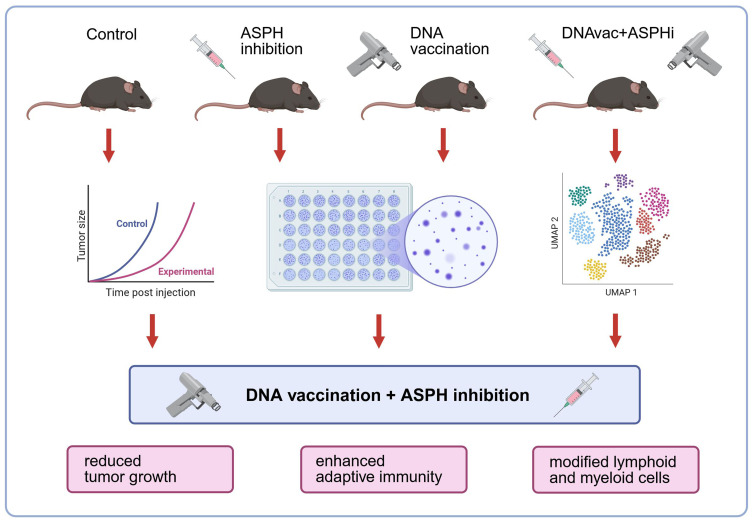

## Introduction

Aspartate β-hydroxylase (ASPH) plays a crucial role in embryonic development by facilitating cell movement for organ formation, but its expression in most normal adult tissues is minimal and localized to the endoplasmic reticulum.^1^,^2^ ASPH is overexpressed in 70–90% of human solid tumors and is associated with poor clinical prognosis in patients with various cancers, making ASPH a valuable prognostic marker.^1–5^ The regulation of ASPH gene expression involves intricate signaling pathways related to cell growth, survival, proliferation, motility, and differentiation, including the insulin/insulin-like growth factor 1 (IGF1)/insulin receptor substrate 1 (IRS1) signaling,^6^ extracellular signal-regulated kinase (ERK)/mitogen-activated protein kinase (MAPK), phosphatidylinositol-3-kinase/protein kinase B (PI3K-Akt) pathways,^7^ and Wnt signaling/β-catenin pathway.^8^ ASPH overexpression increases cell proliferation, motility, and invasion, leading to tumor progression and metastasis.^2^

ASPH catalyzes the hydroxylation of aspartyl and asparaginyl residues in calcium-binding epidermal growth factor (cbEGF)-like domains^2^ and activates numerous oncogenic pathways within tumor cells.^9^ For instance, ASPH hydroxylates Notch1, releasing the Notch intracellular domain (NICD) fragment and promoting cell proliferation and tumor growth.^10–12^ This signaling may be further enhanced by inositol polyphosphate-5-phosphatase F (INPP5F) binding to ASPH.^13^ Additionally, ASPH interacts with vimentin to induce epithelial–mesenchymal transition,^14^ binds pRb to support cell-cycle progression,^15^ stimulates SRC to promote new blood vessel formation,^16^ and regulates glycogen synthase kinase-3β (GSK-3β) to increase cell growth.^17^ Finally, ASPH regulates the expression of Ly6 family members that contribute to tumor development.^18^

Notch signaling also plays a multifaceted role in the development and function of the hematopoietic and immune systems.^19^ This pathway influences cell fate decisions and differentiation processes in both embryogenesis and adult hematopoietic stem cell homeostasis.^19^,^20^ In addition, Notch signaling is crucial in regulating macrophage polarization: Notch activation drives the M1 phenotype independent of M1 or M2 inducers.^21^ Notch proteins are also critical in determining thymocyte development, regulating the choice between T-cell or B-cell lineage commitment, αβ or γδ T-cell receptor expression, and CD4 or CD8 T-cell lineage commitment. In addition, Notch1 plays a role in the activation of peripheral T cells and their differentiation into T helper 1 (Th1), Th2, and regulatory T cell subtypes.^22^ Nevertheless, the ASPH effect on immune cells has only been characterized in natural killer (NK) cells to date.^23^ In that study, NK cells were incubated with recombinant human ASPH, which negatively regulated their activity by increasing apoptosis and necrosis and reducing the surface expression of the natural killer group 2, member D (NKG2D) receptor, and the p44 natural killer cell (NKp44) receptor, and interferon γ (IFN-γ) production. However, the impact on tumor growth was not analyzed.

Small molecule inhibitors (SMIs) have been designed to inhibit the ASPH enzymatic activity^9^ based on the catalytic site at the C-terminus of ASPH.^24^ ASPH inhibition reduced cell proliferation, migration, and invasion in various tumor cell lines, as well as tumor growth and metastasis in several animal models.^9^,^16^,^17^,^24–27^

In addition to NK cells, the effect of ASPH on other immune cells has not been studied. In this study, we determined the impact of ASPH inhibition on both innate and adaptive anti-tumor immune responses and analyzed the phenotypic profiles of immune cells within the spleen and tumor microenvironment (TME). These investigations will provide novel insights into the interplay between ASPH and anti-tumor immunity, potentially revealing new therapeutic strategies targeting ASPH to enhance immune-mediated cancer control.

## Materials and Methods

### Cell Line

The TC-1/A9 cell line (RRID:CVCL_ZW99) used in the study is a derivative of the TC-1 mouse tumor cells (RRID:CVCL_4699) producing the human papillomavirus type 16 (HPV16) E6 and E7 oncoproteins. The TC-1/A9 clone was selected based on suppressed major histocompatibility complex class I (MHC-I) surface expression in a tumor grown in an immunized mouse.^28^ Cells were maintained in Dulbecco’s modified Eagle’s medium (DMEM; Sigma-Aldrich, St. Louis, MO, USA) supplemented with 10% fetal bovine serum (FBS; Sigma-Aldrich), 100 IU/mL penicillin, and 100 μg/mL streptomycin (Biosera, Kansas, MO, USA) and cultured in a humidified incubator at 37°C with a 5% CO_2_ atmosphere. Cells were harvested at 80% confluence using 0.05% trypsin-EDTA in phosphate-buffered saline (PBS) for further experiments.

### Mice

Female 7–8-week-old C57BL/6NCrl mice (Charles River, Sulzfeld, Germany) were used for in vivo experiments, which were performed under specific pathogen-free conditions at the Animal Facility Module of the Czech Center of Phenogenomics (BIOCEV, Vestec, Czech Republic) in compliance with Directive 2010/63/EU. Experimental protocols were approved by the Sectoral Expert Committee of the Czech Academy of Sciences for Approval of Projects of Experiments on Animals (reference number 69/2018, 13 August 2018).

### Cancer Therapy

C57BL/6NCrl mice (n = 10) were subcutaneously (s.c.) inoculated with 3 × 10^4^ TC-1/A9 cells suspended in 0.15 mL PBS (day 0) into the backs under anesthesia with ketamine (100 mg/kg; Bioveta, Ivanovice na Hane, Czech Republic) and xylazine (16 mg/kg; Bioveta). The mice were then treated with 2 μg of the pBSC/PADRE.E7GGG plasmid^29^ by a gene gun (Bio-Rad, Hercules, CA, USA) at a discharge pressure of 400 psi into shaved abdominal skin and/or 50 μg of the ODN1826 adjuvant carrying immunostimulatory CpG motifs (Generi Biotech, Hradec Kralove, Czech Republic) in 200 μL PBS by intraperitoneal (i.p.) injection on days 3, 6, and 10 after cell administration. The dose of 200 μg of the ASPH inhibitor MO-I-1151 in 50 μL DMSO was administered i.p. on days 3–7, 10–14, 17+19+21, 24+26+28, and so on. The empty plasmid pBSC,^30^ PBS, and DMSO were used as controls. Tumor size was measured three times a week with a caliper, and the tumor volume was calculated using the formula (π/6) (a × b × c), where a, b, and c are tumor dimensions.

### Immune Cell Depletion

Subpopulations of immune cells were depleted in vivo by monoclonal antibodies (BioXCell, Lebanon, NH, USA) anti-CD4 (clone GK1.5), anti-CD8 (clone 2.43), and anti-NK1.1 (clone PK136) at doses of 200 μg diluted in 200 μL PBS. These antibodies were administered by i.p. injection on days 4, 7, 11, 14, 18 and 21 after tumor-cell inoculation. PBS was used as a control.

### Flow Cytometry

Single-cell suspensions from tumor tissues or spleens were prepared as previously described.^31^ Cells were stained for viability with the Fixable dye eFluor 506 (ThermoFisher Scientific, Waltham, MA, USA) in PBS, incubated with anti-mouse CD16/32 (Fc block, clone 93; BioLegend, San Diego, CA, USA), and treated with antibodies against surface markers (Table 1). Following fixation and permeabilization (Fixation/Permeabilization Concentrate, eBioscience, San Diego, CA, USA, diluted 1:3 with the Fixation/Permeabilization Diluent, eBioscience), intracellular staining with anti-FoxP3 was performed after staining of lymphoid cells. Flow cytometry data were acquired on a CytoFLEX LX flow cytometer (Beckman Coulter, Indianapolis, IN, USA) and analyzed using FlowJo version 10.8.1 (BD Biosciences, Franklin Lakes, NJ, USA; Figure S1A and B).

**Table 1:** List of Antibodies Used for Flow Cytometry

Antigen	Conjugate	Clone	Company	Staining	Panels	
β2m	AF488	S19.8	Santa Cruz	Surface	•	
CD3	BV421	M1/70	BioLegend	Surface		•
CD4	PerCP-Cy5.5	RM4-5	BioLegend	Surface		•
CD8	FITC	53–6.7	BioLegend	Surface		•
CD11b	BV421	M1/70	BioLegend	Surface	•	
CD11c	APC-Cy7	N418	BioLegend	Surface	•	
CD25	APC	PC61.5	Invitrogen	Surface		•
CD45	AF700	30-F11	BioLegend	Surface	•	•
CD317	PE	927	BioLegend	Surface	•	
F4/80	BV650	BM8	BioLegend	Surface	•	
FoxP3	PE	FJK-16S	Invitrogen	Intracellular		•
Ly6C	BV785	HK1.4	BioLegend	Surface	•	
Ly6G	PE-Cy5	1A8	Reagent Genie	Surface	•	
MHC-II	PE-Cy7	M5/114.15.2	BioLegend	Surface	•	
NK1.1	BV650	PK136	BioLegend	Surface		•
PD-1	PE-Cy7	29F.1A12	BioLegend	Surface		•
TCR γ/δ	BV605	GL3	BioLegend	Surface		•

### ELISPOT Assay

C57BL/6NCrl mice (n = 3) were immunized with 2 μg of the pBSC/PADRE.E7GGG plasmid (group designated DNAvac) using a gene gun on days 0, 3, and 7, inoculated with 200 μg of the ASPH inhibitor MO-I-1151 (ASPHi) in 50 μL DMSO that was injected i. p. on days 0–4, 7–11, and 14, or treated with both the pBSC/PADRE.E7GGG and MO-I-1151 (DNAvac+ASPHi). The empty plasmid pBSC and DMSO were used as controls. On day 15, mononuclear cells were isolated from pooled spleens using a gentleMACS Octo Dissociator (Miltenyi Biotec; m_spleen_4_1 setting), Ficoll/Histopaque (Cytiva, Marlborough, MA, USA) density gradient centrifugation, and CTL-Wash medium (Cellular Technology Limited, Cleveland, OH, USA). The isolated cells were resuspended in CTL-Test medium (Cellular Technology Limited) and incubated overnight with the HPV16 E7-derived peptide (RAHYNIVTF; 0.1 μg/mL; Clonestar Biotech, Brno, Czech Republic) or the PADRE peptide (AKFVAAWTLKAAA; 1 μg/mL; GenScript, Piscataway, NJ, USA) stimulating CD8^+^ or CD4^+^ T cells, respectively. IFN-γ-producing cells were quantified using an ELISPOT assay as previously described.^31^ The stained spots were counted by an ImmunoSpot Analyser S6 Ultimate M2 (Cellular Technology Limited) and relative spot-forming units (SFU) were calculated for each group, with 100% representing the mean SFU obtained after stimulation with the PADRE or E7 peptides in each experiment.

### T-Cell Proliferation Assay

#### Mononuclear Cell Isolation

Spleens from 7–8-week-old female C57BL/6NCrl mice were harvested and dissociated into a single-cell suspension using a gentleMACS Octo Dissociator (m_spleen_01_01 setting), and MACS buffer (consisting of PBS pH 7.2, 0.5% bovine serum albumin (BSA), and 2 mM EDTA). The cells were passed through a 70 µm filter, pelleted by centrifugation at 400×*g* for 10 min at 20°C, and resuspended in 10 mL ACK buffer (0.15 M NH_4_Cl, 10 mM KHCO_3_, 1 mM EDTA, pH 7.2–7.4) for erythrocyte lysis. The cell suspension was again pelleted by centrifugation at 400×*g* for 5 min at 20°C, resuspended in 2 mL PBS, and the numbers of cells were determined.

#### Enrichment of CD3^+^ Cells Using autoMACS

Isolated mononuclear cells were enriched for CD3^+^ cells using the Pan T Cell Isolation Kit II, mouse (Miltenyi Biotec, Gladbach, Germany). Briefly, the counted cells were pelleted by centrifugation at 400×*g* for 5 min at 4°C and resuspended in 40 µL of MACS separation buffer containing 0.5% bovine serum albumin (BSA) in autoMACS Rinsing Solution (Miltenyi Biotec) per 10^7^ total cells, followed by the addition of 10 µL of biotin-antibody cocktail per 10^7^ total cells and incubation for 5 min at 4–8°C. Then, 30 µL of MACS separation buffer and 20 µL of Anti-Biotin MicroBeads per 10^7^ total cells were added, and the cells were incubated for an additional 10 min at 4–8°C. The magnetically labeled cells were then passed through the autoMACS Pro Separator (Miltenyi Biotec) for a standard negative selection (the Depletes program). The number of enriched CD3^+^ T cells in the negative fraction was determined.

#### Cell Staining with CellTrace Violet (CTV)

CD3^+^ T cells were stained using the CellTrace Violet Cell Proliferation Kit (Invitrogen, Waltham, MA, USA) as previously described.^32^ Briefly, after CD3^+^ cells were pelleted (400×*g* for 5 min at 4°C), 9 × 10^6^ cells were resuspended in 3 mL PBS, stained with 3 µL CTV (5 µM), and incubated in a humidified incubator at 37°C in an atmosphere containing 5% CO_2_ for 20 min. The incubation was followed by the addition of 11 mL RPMI-1640 medium (Sigma-Aldrich) supplemented with 100 U/mL penicillin, 100 µg/mL streptomycin, 2 mM L-glutamine, 20 mM HEPES, 0.1% 2-mercaptoethanol, and 10% FBS (all from Sigma-Aldrich) and further incubation at room temperature for 5 min in the dark. Cells were pelleted by centrifugation (400×*g* for 5 min at 4°C) and resuspended in RPMI-1640 at a concentration of 1 × 10^6^/mL.

#### Cell Cultivation

A 96-well U-bottom plate (Techno Plastic Products, Trasadingen, Switzerland) was seeded with 2 × 10^5^ CD3^+^ T cells in 100 μL of RPMI-1640 per well, and the cells were stimulated using a T Cell Activation/Expansion Kit, mouse (Miltenyi Biotec). RPMI-1640 was used as a control. Then, IL-2 (final concentration 100 U/mL; Gibco, Billings, MT, USA) and/or MO-I-1151 (final concentration 10 μM) were added. Cells were cultured in a humidified incubator at 37°C in an atmosphere containing 5% CO_2_ for 24 or 72 hours and then used for flow cytometry analysis.

#### Flow Cytometry

Before staining the cells for flow cytometry, the Anti-Biotin MicroBeads were removed using a SPRIPlate 96R Ring Super Magnet Plate (Beckman Coulter, Indianapolis, IN, USA). Staining was performed using the Fixable Viability Dye eFluor 506 and antibodies against CD4 (PerCP-Cy5.5, clone RM4-5, BioLegend) and CD8 (FITC, clone 53–6.7, BioLegend) and analyzed by FlowJo.

#### Proliferation Index

Lymphocyte proliferation was evaluated by the proliferation index using FlowJo version 10.8.1. It was calculated according to the equation 1.

(1) $$Proliferation Index = {{\mathop \sum \nolimits_1^i {{{G_i} \times i} \over {{2^i}}}} \over {\mathop \sum \nolimits_1^i {{{G_i}} \over {{2^i}}}}}$$

where *i* is the generation index and ${G_i}$ is the number of cells in generation *i*. The proliferation index shows the number of cell divisions while including only cells divided at least once.

### Mouse Tissue Processing for Single-Cell RNA Sequencing (scRNA-Seq)

#### Preparation of Single Cells From Tumors

Single-cell suspensions were prepared from tumors collected 16 days after cell administration using the Tumor Dissociation Kit (Miltenyi Biotec). Briefly, 3 tumors were collected from each group of untreated and treated mice (Control, ASPHi, DNAvac, and DNAvac+ASPHi), and equivalent portions were pooled, cut into pieces less than 3 mm, and transferred to gentleMACS C tubes containing DMEM with enzyme H, enzyme R, and enzyme A according to the manufacturer’s instructions with the addition of 10 μL of 100 μg/mL DNase I (Roche, Basel, Switzerland). Tumor tissue was dissociated into single cells using the gentleMACS Octo Dissociator (programs h_tumor_01_01 and 37C_m_TDK_2) with heaters. The dissociated tissue was passed through a sterile 70 µm filter to obtain a single-cell suspension. After washing, 2 mL of ACK lysing buffer was added to the cell suspension. The single-cell suspensions were centrifuged at 300×*g* for 10 min, and cell number and viability were assessed using AO/EB staining.

#### Enrichment of CD45^+^ Cells by autoMACS

To isolate immune cells from the tumor single-cell suspension, 20 × 10^6^ cells were enriched for CD45^+^ cells using CD45 MicroBeads (Miltenyi Biotec) and magnetic separation by an autoMACS Pro Separator. Briefly, the cell pellet was resuspended in a 180 µL MACS separation buffer, 20 μL CD45 MicroBeads (Miltenyi Biotec) were added, and the cell suspension was incubated at 4°C for 15 min. The cells were then washed by adding 2 mL of MACS separation buffer and centrifuging at 300×*g* for 10 min at 4°C. The cell pellet was resuspended in 100 μL MACS separation buffer and applied to the autoMACS Pro Separator (Miltenyi Biotec) for positive selection using the Possels program. The CD45^+^ cells were collected in a 15 mL tube, and cell number and viability were determined by AO/EB staining.

#### Cell Multiplexing

Enriched CD45^+^ cells at 2 × 10^6^ per sample were pelleted in a 2 mL microcentrifuge tube and resuspended in 1 mL PBS containing 0.04% BSA. The cell suspension was centrifuged at 400×*g* for 5 min at room temperature. The supernatant was removed without disturbing the pellet, and the cells were labeled with Cell Multiplexing Oligo (CMO; 3′ CellPlex Kit Set A; 10x Genomics, Pleasanton, CA, USA) at room temperature with gently pipetting the mixture 5–10 times to resuspend. The mixture was then incubated for 5 min and washed 3 times with PBS containing 10% FBS to remove unbound CMO. After the final wash, assuming 50% cell loss, an appropriate volume of chilled PBS containing 10% FBS was added to the CMO-labeled samples to achieve a final concentration of 700–1,200 cells/μL. Cell concentration and viability were determined using a Countess II Automated Cell Counter (Bio-Rad), and samples were pooled in a ratio of 1:1:1:1. Cell concentration and viability of the pooled sample were determined using a Countess II Automated Cell Counter (Bio-Rad).

#### scRNA-Seq Library Preparation and Sequencing

scRNA-seq libraries were prepared using the Chromium Next GEM Single Cell 3′ Kit v3.1 (10x Genomics) according to the manufacturer’s protocol at the Genomics and Bioinformatics Core Facility, Institute of Molecular Genetics (Prague, Czech Republic). Libraries were sequenced on the Illumina sequencing platform (NovaSeq 6000; Illumina, San Diego, CA, USA) at the Institute of Applied Biotechnologies (Prague, Czech Republic), generating approximately 150 bp paired-end reads.

### scRNA-Seq Data Processing

#### Mapping

Raw data quality was assessed using the FastQC toolkit v0.11.9 (https://www.bioinformatics.babraham.ac.uk/projects/fastqc). Reads were then aligned to the mouse genome, GRCm38 assembly with Ensembl annotation, and counted using the Cell Ranger 6.1.2 (10x Genomics)^33^ pipeline with default parameters. Confident mapping to exonic regions was above 50% for each library. The data are publicly available in the Sequence Read Archive (SRA) database (https://www.ncbi.nlm.nih.gov/sra) under the accession number PRJNA1072571.

#### Sample Quality Control and Clustering

Following Cell Ranger preprocessing, read count data was loaded in R into a Seurat v4.2.1^34^ object. The putative doublets were removed using scDoubletFinder with default parameters,^35^ and low-quality cells were filtered out based on gene expression detection (for tumor samples, 1500–6500 genes and <5% mitochondrial genes). For integration across samples, 3000 highly variable genes were identified (variance stabilization approach implemented in the SCTransform function) and integration anchors were selected (SelectIntegrationFeatures function) based on shared expression patterns. The PrepSCTIntegration function was then used to ensure that all the necessary Pearson residuals were calculated. The integration anchors were predicted using canonical correlation analysis and the SCT test. Finally, the samples were integrated using the IntegrateData function. Dimensionality reduction was performed using principal component analysis (PCA) on the integrated data using the RunPCA function with default parameters. A shared nearest neighbor (SNN) graph^36^ was then built using FindNeighbors over the top 15 principal components (PCs), and cells were clustered using a clustering algorithm based on an SNN modularity optimization implemented in the FindClusters function (resolution: 0.5). Nonlinear dimensionality reduction was performed with uniform manifold approximation and projection (UMAP)^37^ on the top 15 principal components using the RunUMAP function with default parameters. For expression plots and testing of differentially expressed genes, gene counts were log-normalized using the NormalizeData function. Differential expression analysis was performed using the FindAllMarkers function with default parameters.

#### Cluster Annotation

Clusters were annotated using multiple resources including Tabula Muris,^38^ CIPR,^39^ PanglaoDB,^40^ CellKb,^41^ SCSA,^42^ CellMarker,^43^ and AnnotationHub.^44^ We also confirmed cluster annotation with cell markers from differential gene expression analysis.

#### Gene Set and Pathway Enrichment Analyses

The differentially expressed genes were ranked by log fold change, and input to the gene set enrichment analysis (GSEA),^45^ implemented using a custom function built from the clusterProfiler^46^ package v4.8.3 functions, against databases including the Molecular Signatures Database (MSigDB) Hallmark v2020, Kyoto Encyclopedia of Genes and Genomes (KEGG, mouse v2019),^47^ and Gene Ontology (GO)^48^ (Biological Process v2023, Cellular Component v2023, and Molecular Function v2023). The database GMT files were obtained from the Enrichr^49^ and MSigDB^50^ websites. The results were visualized using the ggplot2^48^ R package v3.4.0. Further analysis for cell–cell communication CellChat was performed with default parameters.^51^

### Statistical Analysis

All results were presented as the mean ± the standard error of the mean (SEM). Statistical differences between two groups were determined by the Student’s *t*-test or analysis of variance (ANOVA). All statistical analyses were performed using the Prism software, version 8.4.3 (GraphPad Software, San Diego, CA, USA). *p* < 0.05 was considered statistically significant.

## Results

### Combination of DNA Vaccination and ASPH Inhibition Reduces Tumor Growth

To investigate a possible contribution of ASPH inhibition to cancer immunotherapy, we selected the mouse TC-1/A9 tumor model, which exhibits a low sensitivity to DNA vaccination alone, but tumor growth was significantly reduced after combined immunotherapy with a DNA vaccine and the synthetic oligonucleotide ODN1826^52^ carrying immunostimulatory CpG motifs. Our previous study on ASPH inhibition showed a high ASPH level in TC-1/A9 cells and demonstrated the effect of ASPH inhibition on cell proliferation, migration, and invasion.^18^

**Figure 1:**
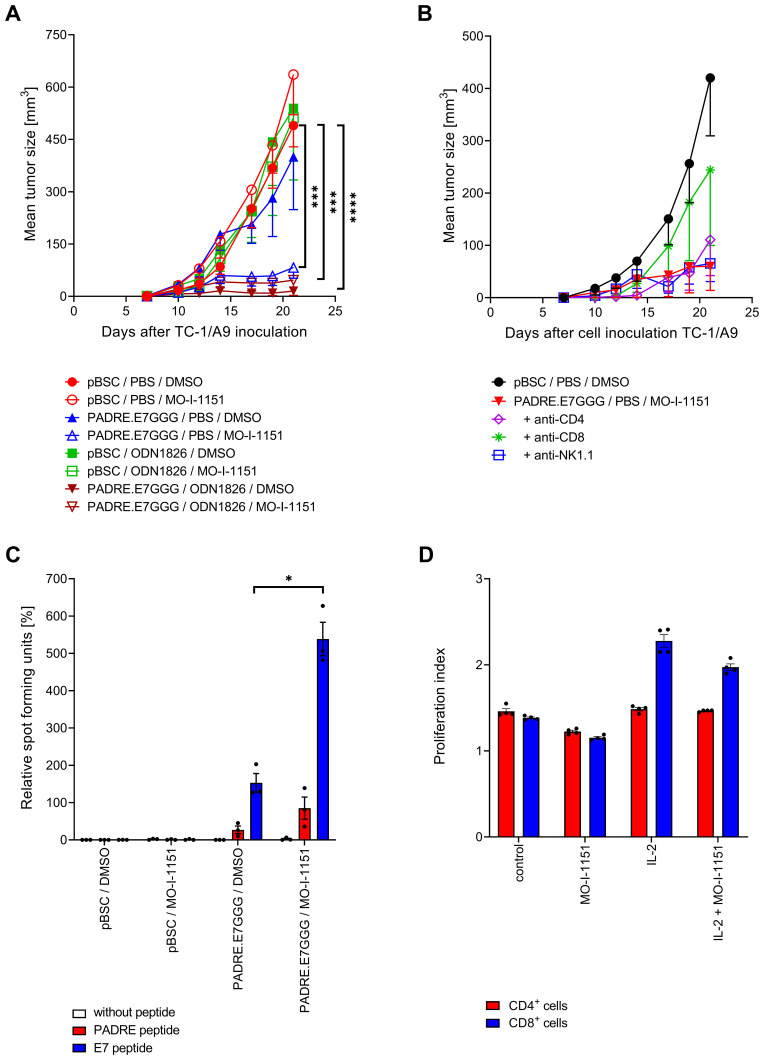
Effect of ASPH inhibition on tumor growth and immune responses. (**A**) Tumor growth. Mice (n=10) were injected s.c. with TC-1/A9 cells and treated with the MO-I-1151 inhibitor, the PADRE.E7GGG DNA vaccine, and the ODN1826 adjuvant alone or in combinations. DMSO, pBSC, and PBS served as respective controls. The experiment was repeated with similar results. The reduced tumor growth after combined therapy with DNA vaccination and ASPH inhibition was also confirmed in in vivo depletion experiments. (**B**) Immune cells involved in tumor growth inhibition. Mice (n=5) were injected s.c. with TC-1/A9 cells and treated with the MO-I-1151 inhibitor and the PADRE.E7GGG DNA vaccine. Subpopulations of immune cells were depleted by i.p. administration of CD4, CD8, or NK1.1 antibodies. Mice treated with pBSC, PBS, and DMSO were used as controls. The experiment was repeated with similar results. (**C**) Specific activation of T cells. Mice (n=3) were treated with the MO-I-1151 inhibitor and/or PADRE.E7GGG DNA vaccine. DMSO and pBSC were used as controls. Mononuclear cells were prepared from pooled spleens, restimulated with peptides, and IFN-γ producing cells were detected by an ELISPOT assay. Columns are the mean of three independent experiments. (**D**) Non-specific proliferation of T cells. CD3^+^ T cells were enriched from splenocytes, labeled with CellTrace Violet, stimulated with CD3ε and CD28 antibodies, and incubated with the MO-I-1151 inhibitor. DMSO was used as a control. IL-2 was added to enhance the proliferation and viability of T cells. T-cell proliferation was analyzed by flow cytometry. Bars, ± SEM; * *p* < 0.05, *** *p* < 0.001, **** *p* < 0.0001.

Here, treating TC-1/A9-induced tumors with the ASPH inhibitor MO-I-1151 alone or in combination with the ODN1826 adjuvant did not significantly reduce tumor growth (Figure 1A). However, combining ASPH inhibition with the PADRE.E7GGG DNA vaccine significantly inhibited tumor growth. This improved anti-tumor effect was not observed when ASPH inhibition was added to the combined treatment by DNA vaccination and ODN1826 administration, which alone showed a strong therapeutic response.

### ASPH Inhibition Enhances the Anti-Tumor Effect of DNA Vaccination by Stimulating the T Cell-Mediated Adaptive Immune Response

To better understand how the MO-I-1151 inhibitor enhanced the anti-tumor effect of DNA vaccination, we performed in vivo depletion of CD4^+^, CD8^+^, or NK1.1^+^ cells using monoclonal antibodies. The anti-tumor effect of the therapy was substantially reduced only by depletion of CD8^+^ cells (Figure 1B). Therefore, we performed an IFN-γ ELISPOT assay to determine whether CD8^+^ T lymphocytes induced by DNA vaccination were further activated after in vivo administration of MO-I-1151. ASPH inhibition increased the number of specifically activated CD8^+^ and CD4^+^ T cells by approximately 3-fold (Figure 1C). We then tested whether enhanced proliferation could account for the increased number of activated T cells. However, the addition of MO-I-1151 to T cells stimulated non-specifically with CD3ε and CD28 antibodies in vitro did not significantly affect the proliferation of either CD8^+^ or CD4^+^ T cells cultivated in the presence or absence of IL-2 (Figure 1D).

### ASPH Inhibition Increases the Reduction of Splenic CD8^+^ T Lymphocytes After DNA Vaccination

**Figure 2:**
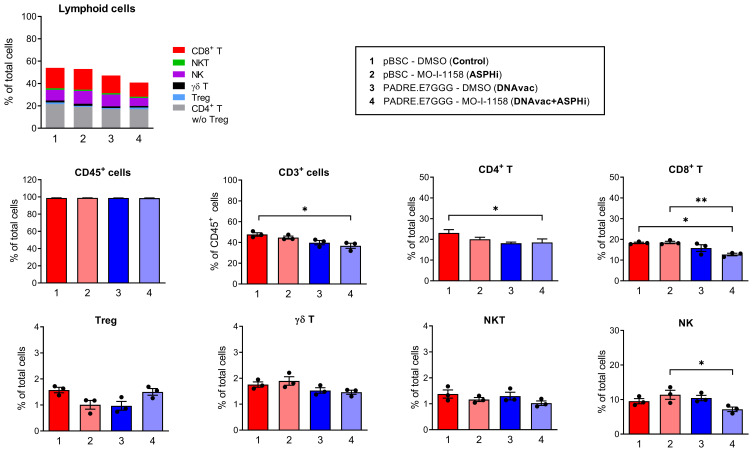
Proportions of T lymphocytes and NK cells in spleens. Mice (n=3) were treated with the MO-I-1151 inhibitor (ASPHi), the PADRE.E7GGG DNA vaccine (DNAvac), or the combination of the ASPH inhibitor and DNA vaccine (DNAvac+ASPHi). Mice treated with DMSO and pBSC were used as controls (Control). Mononuclear cells were isolated from spleens, stained with antibodies, and analyzed by flow cytometry. Bars, ±SEM; * *p* < 0.05, ** *p* < 0.01.

To further analyze immune cells, we used flow cytometry to examine the major subpopulations of T lymphocytes and NK cells in spleens. We compared monocytes isolated from spleens from mice inoculated with the ASPH inhibitor MO-I-1151, immunized with the PADRE.E7GGG DNA vaccine, or treated with the DNA vaccine in combination with the ASPH inhibition (Figure 2). The most abundant cells in all groups were CD4^+^ T lymphocytes, followed by CD8^+^ T cells. DNA vaccination decreased the proportion of T cells, which was most evident for CD8^+^ T cells after the combined treatment DNAvac+ASPHi, when NK cells were also reduced.

### Combination of DNA Vaccination with ASPH Inhibition Reduces Tregs in the Tumor Microenvironment

**Figure 3:**
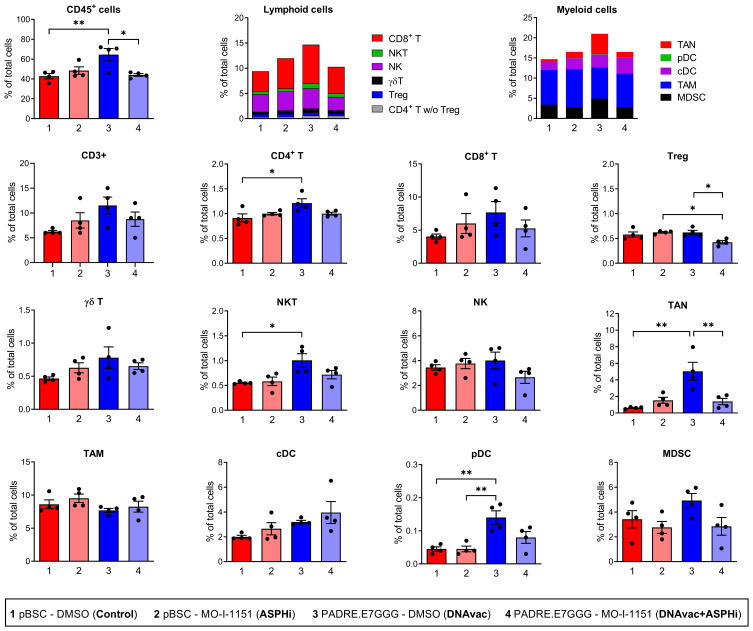
Proportion of immune cells in tumors. Mice (n=4) were injected s.c. with TC-1/A9 cells and treated with the MO-I-1151 inhibitor (ASPHi), the PADRE.E7GGG DNA vaccine (DNAvac), or a combination of the ASPH inhibitor and DNA vaccine (DNAvac+ASPHi). Mice treated with DMSO and pBSC were used as controls (Control). Single cells were isolated from tumors, stained with panels of antibodies for detection of lymphoid and myeloid cells, and analyzed by flow cytometry. Bars, ±SEM; *p < 0.05, **p < 0.01.

We analyzed immune cell infiltration in TC-1/A9-induced tumors treated with an ASPH inhibitor, DNA vaccination, or a combination of both by flow cytometry (Figure 3). DNA vaccination alone significantly increased the infiltration of CD45^+^ immune cells into the tumors compared to untreated tumors. This increase included both lymphoid and myeloid cell lineages. Notably, CD4^+^ T cells (CD45^+^ CD3^+^ γδTCR^−^ NK1.1^−^ CD4^+^), natural killer T (NKT) cells (CD45^+^ CD3^+^ γδTCR^−^, NK1.1^+^), tumor-associated neutrophils (TANs; CD45^+^ CD11b^+^ CD11c^−^ Ly6C^low^ Ly6G^+^ F4/80^−^) and plasmacytoid dendritic cells (pDCs; CD45^+^ CD11c^+^ Ly6G^−^ CD11b^−^ MHC II^+^ F4/80^+^ CD317^+^) were significantly elevated within the TME of DNA-vaccinated mice. Likewise, CD8^+^ T cells (CD45^+^ CD3^+^ γδTCR^−^ NK1.1^−^ CD8^+^), γδ T cells (CD45^+^ CD3^+^ γδTCR^+^), and myeloid-derived suppressor cells (MDSCs; CD45^+^ CD11b^+^ Ly6G^−^ F4/80^−^ Ly6C^hi^) were also increased. Interestingly, the combined DNA vaccination and ASPH inhibition treatment did not enhance the overall CD45^+^ immune cell infiltration – the levels of most immune cell subpopulations after the combined therapy DNAvac+ASPHi were similar to untreated tumors and/or tumors treated with ASPH inhibition alone. We found only a significant reduction of regulatory T cells (Tregs; CD45^+^ CD3^+^ γδTCR^−^ NK1.1^−^ CD4^+^ CD25^+^ Foxp3^+^) and nonsignificant trends toward decrease in NK cells (CD45^+^ CD3^−^ γδTCR^−^ NK1.1^+^) and increase in pDCs and classical dendritic cells (cDCs; CD45^+^ CD11c^+^ F4/80^−^ Ly6G^−^ F4/80^−^ CD317^−^) after combined treatment. These results suggest that an increased level of infiltrating CD8^+^ T cells was not responsible for the anti-tumor effect of the combined therapy DNAvac+ASPHi, but a reduction in the number of Tregs may play a role.

### scRNA-Seq Unveils Immune Diversity Across Therapies

**Figure 4:**
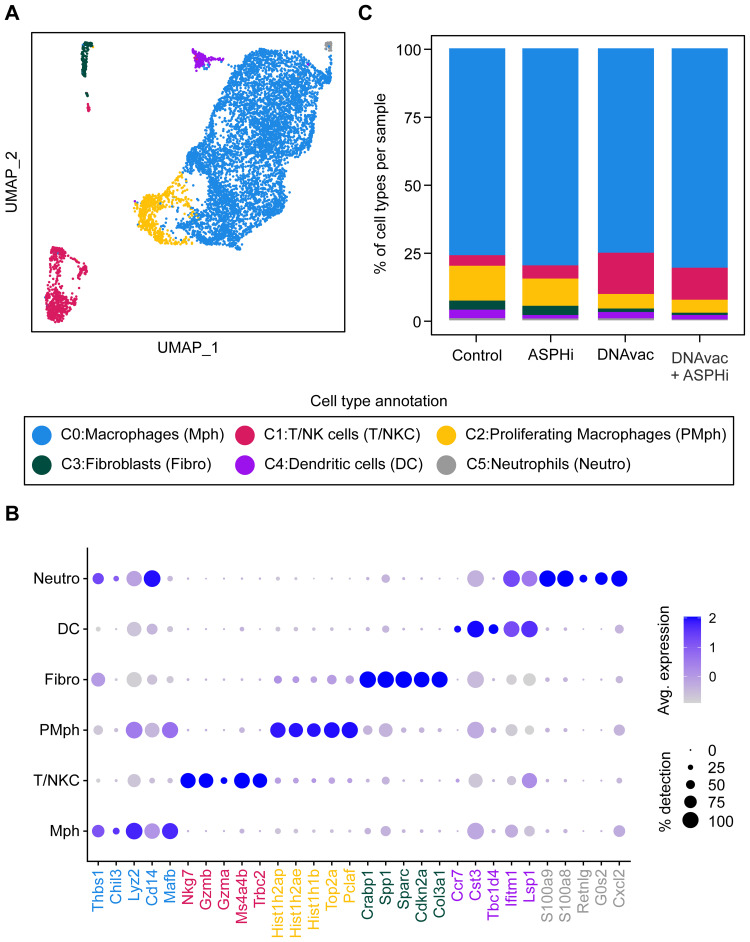
scRNA-seq analysis of tumors. (**A**) The UMAP plot showing 6 major clusters annotated based on bonafide known cell type-specific markers. (**B**) The dot plot representing the top 5 differentially expressed genes in each cluster used to annotate cell types. Dot size indicates the percentage of cells expressing a gene within the cluster, while color represents the relative gene expression level. (**C**) The bar plot representing the composition of cell types in different treatment groups. Bar colors correspond to the cell type clusters in panel (**A**).

scRNA-seq libraries were generated from sorted CD45^+^ tumor-infiltrating immune cells of untreated and treated (ASPHi, DNAvac, and DNAvac+ASPHi) TC-1/A9 tumors, yielding a total of 9734 cells. After filtering out low-quality cells (Figure S2A), 7685 high-quality cells (approximately 1900 cells per sample) were retained for downstream analysis. Unsupervised clustering of these cells identified 6 distinct cellular clusters (Figure 4A) showing unique gene expression profiles (Figure S2B). The expression of cell-type specific markers was used to annotate the obtained clusters (Figures 4B and S2C) which comprised macrophages (*Cd14*), T/NK cells (*Nkg7, Gzmb, Ms4a4b*), proliferating macrophages (*Top2a*), fibroblasts (*Sparc*), dendritic cells (*Ccr7*) and neutrophils (*S100a8/9)*.

The infiltrating immune cells of all integrated tumor samples were predominantly myeloid cells, comprising approximately 90% of the analyzed cell population (Figure 4C; Table S1A). Lymphoid cells were less abundant, representing almost 4% in control tumors and 5% in tumors treated with the ASPH inhibitor alone. Treatment with DNA vaccination led to the highest proportion of lymphoid cells (15%), while the combined therapy DNAvac+ASPHi resulted in a lower lymphocyte infiltration (12%). Interestingly, no B cells were found in the TME of any of the tumor samples. Control and ASPH inhibitor-treated tumors had similar proportions of proliferating macrophages (13% and 10%, respectively) that were reduced to approximately 5% in tumors treated with DNA vaccination alone or combined with ASPH inhibition.

### Combination of DNA Vaccination with ASPH Inhibition Activates Intratumoral CD8^+^ T Cells and NK Cells, and Suppresses Treg Cells

**Figure 5:**
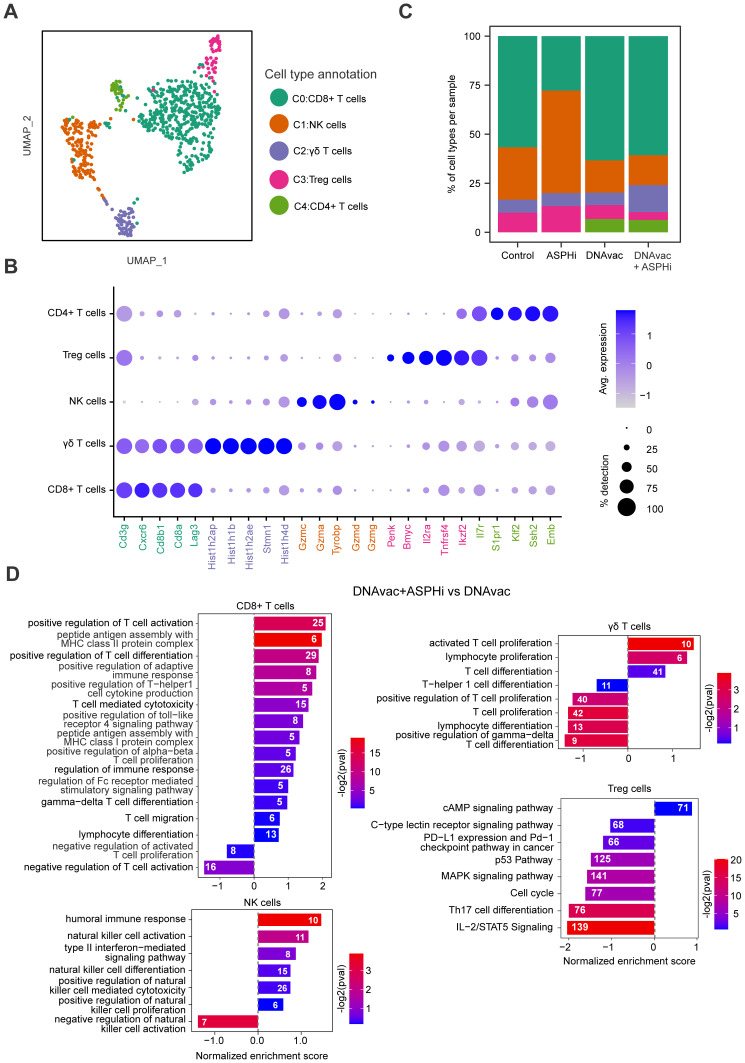
Analysis of T/NK cell subset. (**A**) The UMAP plot representing cellular subclusters. (**B**) The dot plot showing the relative expression of the top 5 cell type-specific markers. Dot size represents the percentage of cells expressing the gene in the target cluster, and color represents the relative average expression of the gene. (**C**) The bar plot showing the relative abundance of different cell populations in four conditions. (**D**) The bar plots representing rank-based normalized enrichment score for the selected pathways in distinct cell populations in DNAvac+ASPHi vs DNAvac comparison. The color scale represents the enrichment *p*-value, numbers in the bars indicate the number of genes enriched in the pathway.

Since we found that the enhancement of anti-tumor immunity by the combined therapy DNAvac+ASPHi was associated with stimulation of T cells, we subset and re-clustered the T/NK cells (cluster 1) to better understand the impact of this combined therapy. This re-clustering revealed five subclusters (Figure 5A) that were annotated using the expression of cell type-specific markers (Figures 5B and S3A). Cell composition analysis (Figure 5C; Table S1B) revealed a slightly higher percentage of CD8^+^ T cells (63.3% vs 60.7%) in tumors treated with DNA vaccination compared to the combined therapy DNAvac+ASPHi. However, a substantial decrease in the proportion of Tregs (4.0% vs 7.1%) and an increase in γδ T cells (13.8% vs 6.4%) were observed in tumors treated with DNA vaccination and ASPH inhibition. In addition, treatment with the ASPH inhibitor alone markedly increased the proportion of NK cells (52.2% vs 26.7% in control tumors) at the expense of CD8^+^ T cells (27.8% vs 56.7%). CD4^+^ T cells were identified only in samples after DNA vaccination alone or combined with ASPH inhibition (approximately 6%).

Each subset exhibited distinct transcriptional changes across conditions, reflecting the impact of the treatments on immune cell activity (Figure S3B). Gene expression was markedly dysregulated in CD8^+^ T cells, γδ T cells, and NK cells after DNAvac and DNAvac+ASPHi treatments compared to control. Changes after ASPH inhibition alone were less prominent except for genes upregulated in NK cells (Figure S3C). NK cells also exhibited differential expression patterns, with ASPHi upregulating genes like *Ccl2,3,4,7, Il15, Il15ra*, and *Klra2*, and DNAvac affecting genes such as *Ccl5, Il23a, and Klrk1*. Treg cells exhibited upregulation of immunoregulatory markers like *Icos, Lag3, Tnfrsf14, Il10, and Cd96* particularly in the DNAvac+ASPHi group, while other genes were downregulated in this group, eg, *Ctla4, Havcr2* (encoding TIM-3), *Tgfb1, FoxP3*, and *Lgals9*. GSEA was performed to investigate the signaling pathways modulated by the DNAvac+ASPHi treatment compared to DNAvac alone (Figure 5D). This analysis highlighted significant alterations of pathways in all T/NK cell subclusters identified in these conditions. While CD8^+^ T-cell, and NK-cell differentiation and function, including cell-mediated cytotoxicity, were stimulated, Treg activity was suppressed after the combination of DNA vaccination with ASPH inhibition. The effect on γδ T cells was ambiguous.

### Cell-to-Cell Communication Between Dendritic Cells and T/NK Cells Is Modified by Combined Treatment with DNA Vaccination and ASPH Inhibition

**Figure 6:**
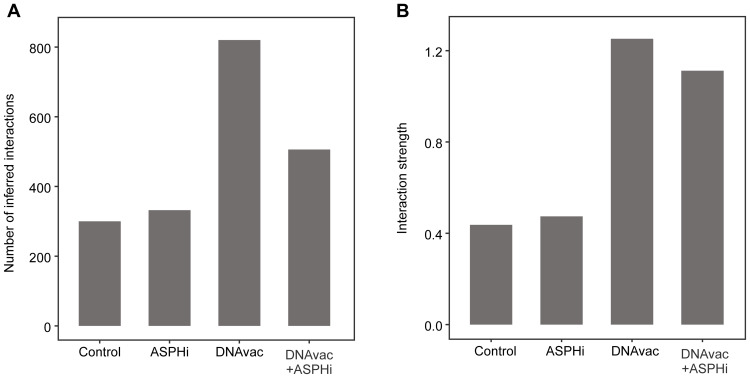
CellChat analysis. (**A**) The bar plot representing the total number of interactions predicted per condition. (**B**) The bar plot showing aggregated interaction strength predicted per conditions.

To understand the interactions between DCs and different T-cell subsets, we predicted expression-based cell-to-cell communication using CellChat. The total number and strength of the interactions in our connectome were highest in the DNAvac group, followed by the DNAvac+ASPHi group (Figure 6).

**Figure 7:**
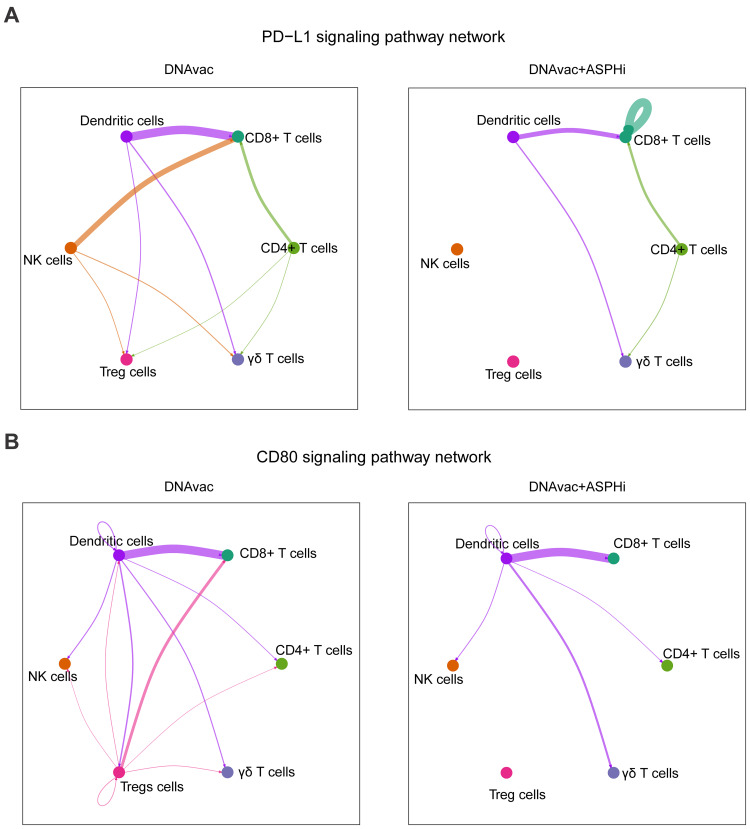
Cell-to-cell communication networks. The connection between dendritic cells and different T/NK cell subsets for (**A**) PD−L1 and (**B**) CD80 signaling pathways was predicted using the CellChat algorithm. The thickness of the edges represents the interaction strength.

The analysis of communication networks of the PD-L1 signaling pathway revealed distinct patterns between DNAvac and DNAvac+ASPHi groups (Figure 7A). In DNAvac, robust and widespread intercellular communication was observed among DCs and lymphoid cells, including NK cells and T cell subpopulations (CD8^+^ T cells, CD4^+^ T cells, γδ T cells, and Treg cells). Notably, CD8^+^ T cells displayed strong incoming signals with DCs, NK cells, and CD4^+^ T cells, whereas no interactions were observed involving Treg cells and γδ T cells. In contrast, DNAvac+ASPHi exhibited a more restricted communication network. The interactions were predominantly confined to DCs, CD8^+^ T cells, and CD4^+^ T cells, with reduced or absent connections involving NK cells, Treg cells, and γδ T cells. Interestingly, a self-signaling loop was observed in CD8^+^ T cells after the combined treatment DNAvac+ASPHi, highlighting a potentially altered autocrine signaling mechanism. Similarly, the communication networks of the CD80 signaling pathway revealed differences in intercellular communication between DNAvac and DNAvac+ASPHi conditions (Figure 7B). In DNAvac, a dense signaling network was observed, with DCs exhibiting strong interactions with CD8^+^ T cells, and weaker interactions with other T/NK cells. In contrast, DNAVac+ASPHi showed a simplified network with particularly a loss of communication between Tregs and all other immune cells. These results suggest more restricted and specific PD-L1 and CD80-mediated communication networks in DNAVac+ASPHi compared to DNAvac treatment.

### Intratumoral Macrophages are Stimulated to Anti-tumor Immune Responses by Combined Treatment with DNA Vaccination and ASPH Inhibition

**Figure 8:**
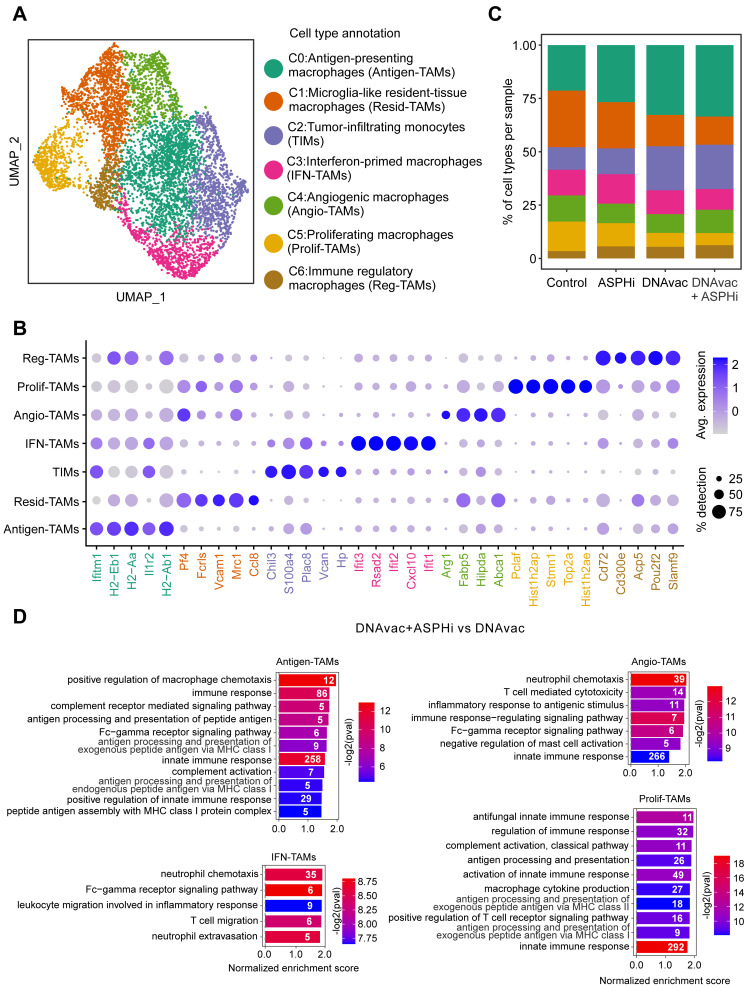
Analysis of macrophage subset. (**A**) The UMAP plot representing cell subclusters. (**B**) The dot plot showing the relative expression of the top 5 cell type-specific markers. Dot size represents the percentage of cells expressing the gene in the target cluster and color represents the relative average expression of the gene. (**C**) The bar plot shows the relative abundance of different cell populations in four conditions. (**D**) The bar plots representing rank-based normalized enrichment score for the selected pathways in different macrophage subpopulations in DNAvac+ASPHi vs DNAvac comparison. The color scale represents the enrichment *p*-value, numbers in the bars indicate the number of genes enriched in the pathway.

We further analyzed the macrophage clusters 0 and 2, finding seven subclusters (Figure 8A) that were annotated using the expression of cell type-specific markers (Figure 8B). The composition of macrophages was significantly altered in the different treatment groups (Figure 2, 8C, Table S1C), particularly antigen-presenting macrophages (Antigen-TAMs), microglia-like resident-tissue macrophages (Resid-TAMs), proliferating macrophages (Prolif-TAMs) and tumor-infiltrating monocytes (TIMs). While Antigen-TAMs and TIMs were increased, Resid-TAMs and Prolif-TAMs were decreased in DNAvac and DNAvac+ASPHi groups compared to Control. The proportions of most macrophage subpopulations were comparable in DNAvac and DNAvac+ASPHi groups. The greatest differences were in angiogenic macrophages (Angio-TAMs; 8.8% vs 10.9%) and interferon-primed macrophages (IFN-TAMs; 11.2% vs 9.7%).

To determine which molecular pathways were altered by the DNAvac+ASPHi therapy compared to DNAvac alone, GSEA was performed using the KEGG database (Figures 8D and S4). The analysis revealed significant pathway alterations in all macrophage subclusters identified under these conditions. In addition to increased levels of Angio-TAMs after the combined therapy DNAvac+ASPHi, these cells exhibited enrichment in the inflammatory response to antigenic stimulus and some other innate immune responses induced by this treatment. The IFN-TAMs showed enrichment in the “Fc-Gamma Receptor Signaling Pathway” and “Leukocyte Migration Involved In Inflammatory Response”, Antigen-TAMs, Resid-TAMs, and Prolif-TAMs were more stimulated in pathways related to complement activation and antigen processing and presentation. The combined therapy DNAvac+ASPHi was also superior in activating chemotaxis in different macrophage types.

## Discussion

ASPH plays a recognized role in tumor initiation and progression in several types of cancer. For this reason, SMIs targeting ASPH have been developed, and their anti-tumor activity has been demonstrated in tumor cell lines and animal models.^9^ However, ASPH inhibition may also affect the immune system, as its targets, particularly Notch receptors, are produced in immune cells. An ASPH effect has already been shown for NK cells in vitro.^23^

Given the growing importance of immunotherapy in cancer treatment, we investigated how ASPH inhibition might influence the anti-tumor immune response in vivo. Our experimental approach used the mouse TC-1/A9 tumor model, which exhibits reversible MHC-I downregulation, resulting in reduced tumor sensitivity to immunotherapy.^28^ In the comparison of 11 mouse tumor models, the TC-1 model has been shown to be a suitable preclinical model of human tumors characterized by poor T-cell infiltration and a high M2/M1 macrophage ratio.^53^ MHC-I downregulation in TC-1/A9 cells, which exhibit a similar infiltration with immune cells to TC-1 cells^54^ further increased the clinical relevance of this model. To explore effects on both innate and adaptive immunity, we combined ASPH inhibition with either immunostimulatory CpG motif (ODN1826) treatment or DNA vaccination targeting the tumor-specific antigen HPV16 E7, respectively. We also tested the ASPH inhibitor alone or in combination with both ODN1826 and a DNA vaccine. The ASPH inhibitor MO-I-1151 alone did not reduce tumor growth, although we found a high level of the ASPH protein in TC-1/A9 cells and demonstrated the effect of ASPH inhibition on cell proliferation, migration, and invasion in our previous in vitro study.^18^ In addition, the dosing regimen successfully inhibited tumor growth and metastasis in other tumor models.^9^,

While treatment with ODN1826 alone or in combination with an ASPH inhibitor did not reduce tumor growth, DNA vaccination showed a modest anti-tumor effect. Importantly, the combination of DNA vaccination with ASPH inhibition significantly enhanced this effect. Further in vivo experiments with the depletion of immune cell subpopulations and analysis of spleen-derived monocytes by an ELISPOT assay supported the hypothesis that ASPH inhibition specifically enhances the adaptive immune response, particularly CD8^+^ T lymphocytes, induced by DNA vaccination. Surprisingly, ASPH inhibition did not further support tumor growth inhibition induced by the potent combination of DNA vaccination and ODN1826 administration. This suggests that the therapeutic benefit of ASPH inhibition on cancer immunotherapy may be context-dependent and less effective when the immune system is already robustly activated. In this case, however, the benefit of ASPH inhibition may still lie in reducing metastasis.

Multiple mechanisms could contribute to the enhanced T-cell response observed with the combined therapy DNAvac+ASPHi. However, flow cytometric analysis of splenocytes and tumor-infiltrating immune cells suggests that the effect of ASPH inhibition is unlikely to be driven by increased T-cell proliferation or tumor infiltration because T-cell proportions in spleens and tumors were rather lower with the combined therapy DNAvac+ASPHi compared to DNA vaccination alone. In addition, in vitro assays confirmed that ASPH did not directly increase T-cell proliferation. This contrasts with the mechanism of the ODN1826-mediated effect on DNA vaccination, which also reduced tumor growth to a similar extent but was associated with a significantly increased CD8^+^ T cell infiltration, as shown in our previous study.^52^

To better understand the molecular changes in immune cells after the combined therapy DNAvac+ASPHi, we performed scRNA-seq on tumor-infiltrating immune cells and confirmatory flow cytometry. Both analyses revealed a lower percentage of total T cells after the combined therapy DNAvac+ASPHi compared to DNA vaccination alone. scRNA-seq also indicated an increase in γδ T cells and a decrease in Tregs in tumor-infiltrating cells, but flow cytometry confirmed only a Treg reduction in tumors. Moreover, the comparison of gene expression in DNAvac and DNAvac+ASPHi groups showed ambiguous effects in stimulating the proliferation and differentiation of γδ T cells. The evaluation of the possible role played by γδ T cells in our experimental system is further complicated by both anti-tumor and pro-tumor effects of these cells.^55^ Next, differential gene expression analysis revealed a higher multifaceted activation of CD8^+^ T cells and NK cells after the combined therapy DNAvac+ASPHi compared to DNA vaccination alone, which was particularly associated with the expression of some activation receptors, chemokine ligands and receptors, and cytokines. Treg cells were not only reduced after the combined therapy DNAvac+ASPHi but also showed different expression profiles. However, because some immunosuppressive markers were downregulated, while others were upregulated, the effect on the functional status of the cells remains unclear, and functional tests are required to clarify this aspect.

The combined therapy DNAvac+ASPHi was also superior in anti-tumor activation of myeloid cells. In different types of macrophages, inflammatory, chemotactic, antigen processing and presentation, complement, and interferon pathways were upregulated more after the combined therapy DNAvac+ASPHi than after DNA vaccination alone. The analysis of cell-to-cell interactions also revealed differences in DC communication with lymphoid cells via PD-L1 and CD80 signaling. Both communication networks were reduced in the combined therapy DNAvac+ASPHi compared to DNA vaccination alone. While the decrease in PD-L1 signaling suggests enhancement of immune responses, the reduced CD80 signaling presents interpretational challenges, warranting careful consideration. These challenges stem from the multifaceted nature of CD80 interactions with different molecules (including CD28, CTLA-4, and PD-L1) both in cis and in trans, either stimulating or inhibiting immune reactions.^56^

These findings suggest that ASPH inhibition in combination with DNA vaccination can enhance the anti-tumor potential of both lymphoid and myeloid immune cells. The primary target cells of ASPH inhibition remain an important area of investigation. Although ASPH production in immune cells is not fully understood, our data provides some clues. scRNA-seq analysis revealed *Asph* expression in most monocytes/macrophages, neutrophils, and DCs, with low levels in lymphocytes. Similarly, the Human Protein Atlas^57^ indicated the highest *ASPH* expression in plasmacytoid DCs and neutrophils in both the HPA and Monaco datasets of bulk RNA-seq. In the scRNA-seq dataset, *ASPH* expression was highest in DCs, slightly lower in macrophages, and markedly lower in lymphocytes. However, the interpretation is complicated by the presence of uncharacterized *ASPH* splicing isoforms. Our preliminary data on mouse splenocytes analyzed by RT-PCR and immunoblotting suggest that the full-length ASPH protein with the enzymatic domain is produced in CD11c^+^ cells, including DCs and macrophages, but not in CD3^+^ T cells. Thus, T cells are probably not the primary target of ASPH inhibition. Their enhanced activity may result from the direct stimulation of DCs and macrophages, which can support anti-tumor immunity by activating CD8^+^ T cells, among other ways.^58^,^59^ Alternatively, ASPH inhibition may indirectly affect immune cells by inhibiting ASPH molecules produced by tumor cells and displayed on their surface^25^ or transferred via exosomes.^11^,^60^ In support of this possibility, the study using recombinant ASPH demonstrated its suppressive effect on NK cell function.^23^

This study introduces new challenges and opportunities in the development of ASPH inhibitors and their potential application in cancer treatment, but some questions remain unanswered. Major limitations of this study include its focus on T cells and limited assessment of other subpopulations of immune cells, as well as analysis of ASPH inhibition combined with DNA vaccination in only one tumor model, which restricts the generalizability of the results. Additionally, the study did not include non-immune cells, nor did it examine the potential systemic immune consequences of ASPH inhibition. Future research should analyze the expression of ASPH isoforms in different immune and non-immune cell types and assess how ASPH inhibition impacts the function of these cells. Furthermore, cancer treatment studies should be conducted in various tumor models that exhibit different levels of MHC-I expression and immunogenicity. The time course of immune responses and the systemic effects of ASPH inhibition should also be investigated.

## Conclusion

We demonstrated that ASPH inhibition enhances anti-tumor immunity induced by DNA vaccination in tumors with reversible downregulation of MHC-I molecules. CD8^+^ T cells were identified as the main effectors of this response, but other immune cells, including NK cells, DCs, and macrophages, could also contribute to the overall response. This broad immunostimulatory effect supports the potential of ASPH inhibition as part of combination therapies for tumors with various levels of MHC-I expression and different mechanisms of MHC-I downregulation.^61–64^ Given its ability to suppress tumor growth and metastasis, ASPH inhibition merits further investigation in clinical trials, particularly in combination with immunotherapy. However, the precise mechanisms by which ASPH inhibition modulates diverse immune cell subsets remain unclear. This lack of mechanistic insight limits our understanding of the underlying pathways and may present challenges in translating these findings to clinical applications. Future studies should aim to elucidate the molecular mechanisms governing immune modulation by ASPH inhibition to fully harness its potential in combination immunotherapy.

## Data Availability

RNA sequencing data generated and analyzed during the current study are available in the Sequence Read Archive under the accession number PRJNA1072571 (https://www.ncbi.nlm.nih.gov/bioproject/PRJNA1072571).

## References

[1] Yang H, Song K, Xue T, et al. The distribution and expression profiles of human aspartyl/asparaginyl beta-hydroxylase in tumor cell lines and human tissues. *Oncol Rep*. 2010;24(5):1257–1264. doi:10.3892/or_00000980 20878118

[2] Zheng W, Wang X, Hu J, Bai B, Zhu H. Diverse molecular functions of aspartate β‑hydroxylase in cancer. *Oncol Rep*. 2020;44(6):2364–2372. doi:10.3892/or.2020.7792 33125119 PMC7610305

[3] Gan X, Li S, Wang Y, et al. Aspartate β-hydroxylase serves as a prognostic biomarker for neoadjuvant chemotherapy in gastric cancer. *Int J Mol Sci*. 2023;24(6):5482. doi:10.3390/ijms24065482 36982561 PMC10053938

[4] Lavaissiere L, Jia S, Nishiyama M, et al. Overexpression of human aspartyl(asparaginyl)beta-hydroxylase in hepatocellular carcinoma and cholangiocarcinoma. *J Clin Invest*. 1996;98(6):1313–1323. doi:10.1172/JCI118918 8823296 PMC507557

[5] Dong X, Lin Q, Aihara A, et al. Aspartate β-hydroxylase expression promotes a malignant pancreatic cellular phenotype. *Oncotarget*. 2015;6(2):1231–1248. doi:10.18632/oncotarget.2840 25483102 PMC4359229

[6] Cantarini MC, de la Monte SM, Pang M, et al. Aspartyl-asparagyl beta hydroxylase over-expression in human hepatoma is linked to activation of insulin-like growth factor and notch signaling mechanisms. *Hepatology*. 2006;44(2):446–457. doi:10.1002/hep.21272 16871543

[7] Hou G, Xu B, Bi Y, et al. Recent advances in research on aspartate β-hydroxylase (ASPH) in pancreatic cancer: a brief update. *Bosn J Basic Med Sci*. 2018;18(4):297–304. doi:10.17305/bjbms.2018.3539 30179586 PMC6252103

[8] Moon RT, Kohn AD, De Ferrari GV, Kaykas A. WNT and beta-catenin signalling: diseases and therapies. *Nat Rev Genet*. 2004;5(9):691–701. doi:10.1038/nrg1427 15372092

[9] Kanwal M, Smahel M, Olsen M, Smahelova J, Tachezy R. Aspartate β-hydroxylase as a target for cancer therapy. *J Exp Clin Cancer Res*. 2020;39(1):163. doi:10.1186/s13046-020-01669-w 32811566 PMC7433162

[10] Dinchuk JE, Focht RJ, Kelley JA, et al. Absence of post-translational aspartyl β-hydroxylation of epidermal growth factor domains in mice leads to developmental defects and an increased incidence of intestinal neoplasia. *J Biol Chem*. 2002;277(15):12970–12977. doi:10.1074/jbc.M110389200 11773073

[11] Lin Q, Chen X, Meng F, et al. ASPH-notch axis guided exosomal delivery of prometastatic secretome renders breast cancer multi-organ metastasis. *Mol Cancer*. 2019;18(1):156. doi:10.1186/s12943-019-1077-0 31694640 PMC6836474

[12] Yuan X, Wu H, Xu H, et al. Notch signaling: an emerging therapeutic target for cancer treatment. *Cancer Lett*. 2015;369(1):20–27. doi:10.1016/j.canlet.2015.07.048 26341688

[13] Zhou Y, Yuan Y, Zhang Q, Shen Y, Chen W, Yan L. Downregulation of SLC14A1 expression indicates poor prognosis and promotes the progression of non-small cell lung cancer. *Ann Clin Lab Sci*. 2022;52(5):753–762. 36261188

[14] Zou Q, Hou Y, Wang H, et al. Hydroxylase activity of ASPH promotes hepatocellular carcinoma metastasis through epithelial-to-mesenchymal transition pathway. *EBioMedicine*. 2018;31:287–298. doi:10.1016/j.ebiom.2018.05.004 29764768 PMC6013968

[15] Huang C-K, Iwagami Y, Zou J, et al. Aspartate beta-hydroxylase promotes cholangiocarcinoma progression by modulating RB1 phosphorylation. *Cancer Lett*. 2018;429:1–10. doi:10.1016/j.canlet.2018.04.041 29733964 PMC5985220

[16] Ogawa K, Lin Q, Li L, et al. Aspartate β-hydroxylase promotes pancreatic ductal adenocarcinoma metastasis through activation of SRC signaling pathway. *J Hematol Oncol*. 2019;12(1):144. doi:10.1186/s13045-019-0837-z 31888763 PMC6937817

[17] Iwagami Y, Huang C-K, Olsen MJ, et al. Aspartate β-hydroxylase modulates cellular senescence through glycogen synthase kinase 3β in hepatocellular carcinoma. *Hepatology*. 2016;63(4):1213–1226. doi:10.1002/hep.28411 26683595 PMC4805474

[18] Kanwal M, Smahelova J, Ciharova B, et al. Aspartate β-hydroxylase regulates expression of Ly6 genes. *J Cancer*. 2024;15(5):1138–1152. doi:10.7150/jca.90422 38356711 PMC10861829

[19] Radtke F, Fasnacht N, Macdonald HR. Notch signaling in the immune system. *Immunity*. 2010;32(1):14–27. doi:10.1016/j.immuni.2010.01.004 20152168

[20] Radtke F, MacDonald HR, Tacchini-Cottier F. Regulation of innate and adaptive immunity by Notch. *Nat Rev Immunol*. 2013;13(6):427–437. doi:10.1038/nri3445 23665520

[21] Wang Y-C, He F, Feng F, et al. Notch signaling determines the M1 versus M2 polarization of macrophages in antitumor immune responses. *Cancer Res*. 2010;70(12):4840–4849. doi:10.1158/0008-5472.CAN-10-0269 20501839

[22] Osborne BA, Minter LM. Notch signalling during peripheral T-cell activation and differentiation. *Nat Rev Immunol*. 2007;7(1):64–75. doi:10.1038/nri1998 17170755

[23] Huyan T, Li Q, Ye LJ, et al. Inhibition of human natural killer cell functional activity by human aspartyl β-hydroxylase. *Int Immunopharmacol*. 2014;23(2):452–459. doi:10.1016/j.intimp.2014.09.018 25281391

[24] Brewitz L, Tumber A, Thalhammer A, Salah E, Christensen KE, Schofield CJ. Synthesis of novel pyridine-carboxylates as small-molecule inhibitors of human aspartate/asparagine-β-hydroxylase. *ChemMedChem*. 2020;15(13):1139–1149. doi:10.1002/cmdc.202000147 32330361 PMC7383925

[25] Aihara A, Huang CK, Olsen MJ, et al. A cell-surface β-hydroxylase is a biomarker and therapeutic target for hepatocellular carcinoma. *Hepatology*. 2014;60(4):1302–1313. doi:10.1002/hep.27275 24954865 PMC4176525

[26] Huang CK, Iwagami Y, Aihara A, et al. Anti-tumor effects of second generation β-hydroxylase inhibitors on cholangiocarcinoma development and progression. *PLoS One*. 2016;11(3):e0150336. doi:10.1371/journal.pone.0150336 26954680 PMC4783022

[27] Nagaoka K, Ogawa K, Ji C, et al. Targeting aspartate beta-hydroxylase with the small molecule inhibitor MO-I-1182 suppresses cholangiocarcinoma metastasis. *Dig Dis Sci*. 2021;66(4):1080–1089. doi:10.1007/s10620-020-06330-2 32445050

[28] Smahel M, Síma P, Ludvíková V, Marinov I, Pokorná D, Vonka V. Immunisation with modified HPV16 E7 genes against mouse oncogenic TC-1 cell sublines with downregulated expression of MHC class I molecules. *Vaccine*. 2003;21(11–12):1125–1136. doi:10.1016/s0264-410x(02)00519-4 12559790

[29] Smahel M, Polakova I, Duskova M, Ludvikova V, Kastankova I. The effect of helper epitopes and cellular localization of an antigen on the outcome of gene gun DNA immunization. *Gene Ther*. 2014;21(2):225–232. doi:10.1038/gt.2013.81 24385146

[30] Smahel M, Síma P, Ludvíková V, Vonka V. Modified HPV16 E7 genes as DNA vaccine against E7-containing oncogenic cells. *Virology*. 2001;281(2):231–238. doi:10.1006/viro.2000.0794 11277695

[31] Kaštánková I, Poláková I, Dušková M, Šmahel M. Combined cancer immunotherapy against Aurora kinase A. *J Immunother*. 2016;39(4):160–170. doi:10.1097/CJI.0000000000000120 27070447

[32] Grosjean C, Quessada J, Nozais M, Loosveld M, Payet-Bornet D, Mionnet C. Isolation and enrichment of mouse splenic T cells for and T cell receptor stimulation assays. *STAR Protoc*. 2021;2(4):100961. doi:10.1016/j.xpro.2021.100961 34825221 PMC8605083

[33] Zheng GXY, Terry JM, Belgrader P, et al. Massively parallel digital transcriptional profiling of single cells. *Nat Commun*. 2017;8:14049. doi:10.1038/ncomms14049 28091601 PMC5241818

[34] Hao Y, Hao S, Andersen-Nissen E, et al. Integrated analysis of multimodal single-cell data. *Cell*. 2021;184(13):3573–3587.e29. doi:10.1016/j.cell.2021.04.048 34062119 PMC8238499

[35] Germain PL, Lun A, Garcia Meixide C, Macnair W, Robinson MD. Doublet identification in single-cell sequencing data using scDblFinder. *F1000Res*. 2022;10:979. doi:10.12688/f1000research.73600.2 PMC9204188 35814628

[36] Ertöz L, Steinbach M, Kumar V. Finding clusters of different sizes, shapes, and densities in noisy, high dimensional data. In: Proceedings of the 2003 SIAM International Conference on Data Mining. Society for Industrial and Applied Mathematics; 2003:47–58. doi:10.1137/1.9781611972733.5.

[37] McInnes L, Healy J, Melville J. UMAP: uniform manifold approximation and projection for dimension reduction. *arXiv*. 2020. doi:10.48550/arXiv.1802.03426

[38] Tabula Muris Consortium, Overall coordination, Logistical coordination, et al. Single-cell transcriptomics of 20 mouse organs creates a Tabula Muris. *Nature*. 2018;562(7727):367–372. doi:10.1038/s41586-018-0590-4 30283141 PMC6642641

[39] Ekiz HA, Conley CJ, Stephens WZ, O’Connell RM. CIPR: a web-based R/shiny app and R package to annotate cell clusters in single cell RNA sequencing experiments. *BMC Bioinformatics*. 2020;21(1):191. doi:10.1186/s12859-020-3538-2 32414321 PMC7227235

[40] Franzén O, Gan LM, Björkegren JLM. PanglaoDB: a web server for exploration of mouse and human single-cell RNA sequencing data. *Database*. 2019;2019. doi:10.1093/database/baz046 PMC6450036 30951143

[41] Patil A, Patil A. CellKb Immune: a manually curated database of mammalian hematopoietic marker gene sets for rapid cell type identification. *bioRxiv*. 2020. doi:10.1101/2020.12.01.389890

[42] Cao Y, Wang X, Peng G. SCSA: a cell type annotation tool for single-cell RNA-seq data. *Front Genet*. 2020;11:490. doi:10.3389/fgene.2020.00490 32477414 PMC7235421

[43] Zhang X, Lan Y, Xu J, et al. CellMarker: a manually curated resource of cell markers in human and mouse. *Nucleic Acids Res*. 2019;47(D1):D721–D728. doi:10.1093/nar/gky900 30289549 PMC6323899

[44] Morgan M, Shepherd L. AnnotationHub: client to access AnnotationHub resources. *Bioconductor*. 2024. doi:10.18129/B9.bioc.AnnotationHub

[45] Subramanian A, Tamayo P, Mootha VK, et al. Gene set enrichment analysis: a knowledge-based approach for interpreting genome-wide expression profiles. *Proc Natl Acad Sci USA*. 2005;102(43):15545–15550. doi:10.1073/pnas.0506580102 16199517 PMC1239896

[46] Yu G, Wang LG, Han Y, He QY. clusterProfiler: an R package for comparing biological themes among gene clusters. *OMICS*. 2012;16(5):284–287. doi:10.1089/omi.2011.0118 22455463 PMC3339379

[47] Kanehisa M, Goto S. KEGG: Kyoto encyclopedia of genes and genomes. *Nucleic Acids Res*. 2000;28(1):27–30. doi:10.1093/nar/28.1.27 10592173 PMC102409

[48] Wickham H. Ggplot2. *Wiley Interdiscip Rev Comput Stat*. 2011;3(2):180–185. doi:10.1002/wics.147

[49] Kuleshov MV, Jones MR, Rouillard AD, et al. Enrichr: a comprehensive gene set enrichment analysis web server 2016 update. *Nucleic Acids Res*. 2016;44(W1):W90–7. doi:10.1093/nar/gkw377 27141961 PMC4987924

[50] Liberzon A, Subramanian A, Pinchback R, Thorvaldsdóttir H, Tamayo P, Mesirov JP. Molecular signatures database (MSigDB) 3.0. *Bioinformatics*. 2011;27(12):1739–1740. doi:10.1093/bioinformatics/btr260 21546393 PMC3106198

[51] Jin S, Guerrero-Juarez CF, Zhang L, et al. Inference and analysis of cell-cell communication using CellChat. *Nat Commun*. 2021;12(1):1088. doi:10.1038/s41467-021-21246-9 33597522 PMC7889871

[52] Grzelak A, Polakova I, Smahelova J, et al. Experimental combined immunotherapy of tumours with major histocompatibility complex class I downregulation. *Int J Mol Sci*. 2018;19(11):3693. doi:10.3390/ijms19113693 30469401 PMC6274939

[53] Sprooten J, Vanmeerbeek I, Datsi A, et al. Lymph node and tumor-associated PD-L1+ macrophages antagonize dendritic cell vaccines by suppressing CD8+ T cells. *Cell Rep Med*. 2024;5(1):101377. doi:10.1016/j.xcrm.2023.101377 38232703 PMC10829875

[54] Lhotakova K, Grzelak A, Polakova I, Vackova J, Smahel M. Establishment and characterization of a mouse tumor cell line with irreversible downregulation of MHC class I molecules. *Oncology Rep*. 2019;42(6):2826–2835. doi:10.3892/or.2019.7356 31638243

[55] Park JH, Lee HK. Function of γδ T cells in tumor immunology and their application to cancer therapy. *Exp Mol Med*. 2021;53(3):318–327. doi:10.1038/s12276-021-00576-0 33707742 PMC8080836

[56] Li L, Yang L, Jiang D. Research progress of CD80 in the development of immunotherapy drugs. *Front Immunol*. 2024;15:1496992. doi:10.3389/fimmu.2024.1496992 39575257 PMC11578925

[57] Uhlen M, Oksvold P, Fagerberg L, et al. Towards a knowledge-based Human Protein Atlas. *Nat Biotechnol*. 2010;28(12):1248–1250. doi:10.1038/nbt1210-1248 21139605

[58] Fu C, Jiang A. Dendritic cells and CD8 T cell immunity in tumor microenvironment. *Front Immunol*. 2018;9:3059. doi:10.3389/fimmu.2018.03059 30619378 PMC6306491

[59] Xiao Z, Wang R, Wang X, et al. Impaired function of dendritic cells within the tumor microenvironment. *Front Immunol*. 2023;14:1213629. doi:10.3389/fimmu.2023.1213629 37441069 PMC10333501

[60] Ogawa K, Lin Q, Li L, et al. Prometastatic secretome trafficking via exosomes initiates pancreatic cancer pulmonary metastasis. *Cancer Lett*. 2020;481:63–75. doi:10.1016/j.canlet.2020.02.039 32145343 PMC7309190

[61] de Vries NL, van de Haar J, Veninga V, et al. γδ T cells are effectors of immunotherapy in cancers with HLA class I defects. *Nature*. 2023;613(7945):743–750. doi:10.1038/s41586-022-05593-1 36631610 PMC9876799

[62] Han J, Dong L, Wu M, Ma F. Dynamic polarization of tumor-associated macrophages and their interaction with intratumoral T cells in an inflamed tumor microenvironment: from mechanistic insights to therapeutic opportunities. *Front Immunol*. 2023;14:1160340. doi:10.3389/fimmu.2023.1160340 37251409 PMC10219223

[63] Beck JD, Diken M, Suchan M, et al. Long-lasting mRNA-encoded interleukin-2 restores CD8+ T cell neoantigen immunity in MHC class I-deficient cancers. *Cancer Cell*. 2024;42(4):568–582.e11. doi:10.1016/j.ccell.2024.02.013 38490213

[64] Lerner EC, Woroniecka KI, D’Anniballe VM, et al. CD8+ T cells maintain killing of MHC-I-negative tumor cells through the NKG2D–NKG2DL axis. *Nat Cancer*. 2023;4(9):1258–1272. doi:10.1038/s43018-023-00600-4 37537301 PMC10518253

